# CKD Care Programs and Incident Kidney Failure: A Study of a National Disease Management Program in Taiwan

**DOI:** 10.1016/j.xkme.2022.100485

**Published:** 2022-05-21

**Authors:** Ming-Yen Lin, Yi-Wen Chiu, Yung-Ho Hsu, Mai-Szu Wu, Jer-Ming Chang, Chih-Cheng Hsu, Chih-Wei Yang, Wu-Chang Yang, Shang-Jyh Hwang

**Affiliations:** 1 Division of Nephrology, Department of Internal Medicine, Kaohsiung Medical University Hospital, Kaohsiung Medical University, Kaohsiung, Taiwan; 2 Department of Renal Care, College of Medicine, Kaohsiung Medical University, Kaohsiung, Taiwan; 3 Division of Nephrology, Department of Internal Medicine, School of Medicine, College of Medicine, Taipei Medical University, Taipei, Taiwan; 4 Division of Nephrology, Taipei Medical University-Hsin Kuo Min Hospital, Taoyuan, Taiwan; 5 Division of Nephrology, Taipei Medical University-Shuang Ho Hospital, Taipei, Taiwan; 6 Institute of Population Health Sciences, National Health Research Institutes, Miaoli, Taiwan; 7 Department of Nephrology, Chang Gung Memorial Hospital, College of Medicine, Chang Gung University, Taoyuan, Taiwan; 8 Division of Nephrology, Landseed International Hospital, Taoyuan 32001, Taiwan; 9 Program in Toxicology, College of Pharmacy, Kaohsiung Medical University, Kaohsiung, Taiwan

**Keywords:** Chronic kidney disease care, dialysis duration, end-stage kidney disease, incidence, kidney failure, maintenance dialysis, mortality, prevalence, secular trends, universal care

## Abstract

**Rationale & Objective:**

Taiwan implemented national pay-for-performance programs for chronic kidney disease (CKD) care in 2006 and 2011; however, it is unknown whether this affected trends in maintenance dialysis. This study assessed the temporal trends in the incidence, prevalence, and mortality of individuals treated with maintenance dialysis from 2002-2016 in Taiwan.

**Study Design:**

Follow-up study using Taiwan Renal Disease System Databases.

**Setting & Participants:**

Participants who received dialysis for ≥90 days.

**Predictors:**

Age, sex, and calendar year.

**Outcomes:**

Incidence, prevalence of maintenance dialysis, or death, ascertained using the National Death Registry database.

**Analytical Approach:**

The estimated annual percentage change was assessed by a generalized linear model, and the association of the programs with changes in the incidence of maintenance dialysis was evaluated using an age-period-cohort model.

**Results:**

A total of 144,258 incident cases with a follow-up of 346 million person-years were analyzed during the observed periods. The estimated annual percentage change of the expected crude incidence rate was slightly reduced by 0.41% (95% CI, −1.06 to 0.24) and was more obvious in women and patients aged greater than 70 years; whereas, it was significantly increased in those aged greater than 75 years. After disentangling age and cohort effects, the implementation of the care programs was associated with an overall net drift of −1.09% (95% CI, −1.65 to −0.52) per year and a significant linear reduction in the period rate ratio from 1.06 (95% CI, 1.02-1.09) in the years 2002-2006 to 0.95 (95% CI, 0.92-0.98) in 2012-2016, using years 2007-2011 as reference.

**Limitations:**

The findings of the study may have limited inferences to other countries with different health care systems.

**Conclusions:**

The implementation of universal CKD care programs in Taiwan has significantly reduced the long-term trends in the incidence of maintenance dialysis; hence, devoting governmental resources to CKD care and prevention is advocated.

## Graphical abstract

**Figure:**
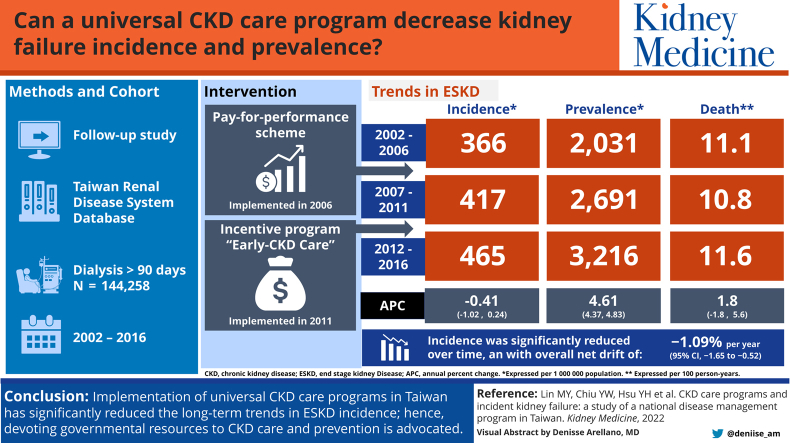

**Plain-Language Summary**

Chronic kidney disease (CKD) care is a central strategy to delay dialysis and avoid early mortality. It is unknown how 1 country’s implementation of universal CKD care programs can affect long-term trends of the incidence of kidney failure. We assessed net trends of the incidence of kidney failure across the CKD care programs implemented and observed an almost 10% annual net reduction in the incidence rate of kidney failure from 2002-2016 in Taiwan before and after the national incentivized CKD care policies were implemented. Although the young population exhibited declining trends, the elderly population exhibited rising trends. Although CKD care affects kidney failure incidence rates with varying scales, CKD care and prevention should be prioritized by countries to reduce the growing numbers of kidney failure cases.

Global cases of individuals treated with maintenance dialysis are dramatically rising because of improved life expectancy and the development of kidney replacement therapy (KRT). It is estimated that the worldwide number of patients who maintain their life using KRT will double to 5.439 million in 2030 from 2.618 million in 2010.^1^ To effectively reduce the disease burden of kidney failure treated with maintenance dialysis, the concept of chronic kidney disease (CKD) was defined by the NKF-KDOQI (National Kidney Foundation–Kidney Disease Outcomes Quality Initiative) guidelines in 2002.^2^ According to the principles mentioned within the guidelines, many countries developed their strategies for the early prevention and detection of CKD, KRT modality choice, and conservative care rather than KRT during the past 20 years to respond to the huge challenges of the rapidly increasing population receiving maintenance dialysis.^3^ In general, public awareness, communications, and responses to CKD across health specialties and between health care providers and patients have largely improved. However, the influences of a national kidney prevention policy on the long-term trend of maintenance dialysis are less well-studied.

In 2006, Taiwan National Health Insurance launched a pay-for-performance scheme with indicators for early CKD care to encourage nephrologists to cooperate with nurses and dietitians to provide comprehensive care on the basis of the NKF-KDOQI guidelines for patients with an estimated glomerular filtration rate of <45 mL/min/1.73 m^2^ or severe proteinuria (urine protein creatinine ratio > 1,000 mg/g). Health care workers could obtain additional bonuses once they have comprehensively provided patient care and education to improve kidney function progression and dialysis preparation.^4^ According to the Taiwan Renal Data System, the program has covered over half (55%-63%) of the patients who started long-term dialysis treatment from 2013-2018.^5^ To extend the preventive concept to an earlier stage and to specialists other than nephrologists, another care incentive program named Early-CKD Care was launched in 2011 for patients whose estimated glomerular filtration rate was between 45 and 59 mL/min/1.73 m^2^ and for those whose estimated glomerular filtration rate was ≥60 mL/min/1.73 m^2^ with accompanying proteinuria. The Early-CKD Care program paid an additional bonus for timely patient enrollment, regular evaluation, and nephrology referral. Physicians other than nephrologists could be reimbursed for early CKD care after being trained and certified by the Taiwan Society of Nephrology.^4^

The long-term changes in the trends of kidney failure and earlier stages of CKD in different countries have been routinely declared and compared in the US Renal Data System annual report.^6^ By collecting data on kidney failure and earlier stages of CKD from countries around the world, people can study and infer the factors influencing changes in the long-term trends of CKD. However, clarifying the effects of national policy on the long-term trends of kidney disease in a country that lacks a comprehensive kidney disease registry is quite challenging. Taiwan introduced a mandatory, universal, single-payer insurance system in 1995, with all dialysis therapies and kidney transplantation care only reimbursed by National Health Insurance without copayment from patients requiring KRT. Therefore, exploring long-term changes in disease incidence, prevalence, and mortality is more appropriate and less biased.

Age-period-cohort analysis has been used to understand disease trends by attempting to disentangle the factors that influence all ages (period effects), such as changes in health policy, from those that vary by generation (cohort effects), typically as a consequence of common exposure. To our knowledge, there is no study describing the change in maintenance dialysis trends after universal CKD health policy implementation. We aimed to conduct a retrospective population-based study to explore the long-term trend of maintenance dialysis variations in incidence, prevalence, average dialysis duration, and mortality in Taiwan during 2002-2016.

## Methods

### Study Design and Data Sources

This study used the 2002-2016 summary data from the latest Annual Report of Kidney Disease in Taiwan by the Taiwan Data Renal Report System. The Taiwan Data Renal Report System is mainly composed of the National End-Stage Kidney Disease Registry database, Taiwan’s National Health Insurance Research Database,^7^ National Death Registry database, Taiwan Organ Registry database, and Population Census Database.^8^ To distribute information on kidney disease in Taiwan, the Taiwan Data Renal Report System committee annually provides accurate statistics to the US Renal Data System and has published a report since 2014.^9^ The analyzed data are annually updated over time through formally applying to the National Health Insurance Administration. This study was approved by the Institutional Review Board of Kaohsiung Medical University Hospital (KMUHIRB-EXEMPT(I)-20200021). The requirement for informed consent was waived by the Ethical Review Board because the study used summarized data from public sources. All study procedures were conducted according to the principles of the Declaration of Helsinki.

### Maintenance Dialysis Treatment and Mortality

Patients with kidney failure who were treated by dialysis for ≥90 days were identified by the reimbursed codes for dialysis therapy (Table S1) from the National Health Insurance Research Database. The dates of the first dialysis were retrieved from the first appearance of each patient’s specific reimbursed code. Because only a few patients with kidney failure receive kidney transplantation before dialysis in Taiwan, they were not included in the main analyses. Death and dates of death in the population receiving dialysis were ascertained by examining the National Death Registry data.

### Statistical Analysis

The yearly incidence, prevalence, and mortality of maintenance dialysis by different age and sex groups were obtained from the Appendix of the 2019 Annual Report on Kidney Disease in Taiwan.^9^ Age was categorized into 5-year intervals, 0-4, 5-9…80-84, and greater than 85 years in the overall group and according to sex. To detect a change in the trend, the years were separated into 3 periods, 2002-2006, 2007-2011, and 2012-2016, to reflect temporal variation. Through the age and period categories, 20 birth cohorts with 5-year intervals, 1917-1921, 1922-1926, …2007-2011, and 2012-2016, were generated simultaneously. Period incidence rates were obtained by dividing the sum of all new maintenance dialysis cases in 1 period by summing the mid-year population estimates of each year in the period and multiplying the result by 1,000,000. The annual prevalence proportions were calculated by dividing the number of individuals treated with maintenance dialysis at the end of each year by population size and multiplying the result by 1,000,000. The average annual prevalence is presented for 2002-2006, 2007-2011, and 2012-2016. Mortality rates of maintenance dialysis were calculated by dividing the sum of mortality numbers of maintenance dialysis in 1 period by summing up the mid-year maintenance dialysis estimates of each year in the period and multiplying the result by 100. The period average durations of dialysis were calculated by the ratio of prevalence proportion in 1 period to the incidence rate. To identify the influences of age on maintenance dialysis trends, we stratified all estimations by age in the overall population and according to sex. The age-standardized incidence rates (ASRs) were calculated from the age distribution of the World Health Organization’s 2000 standard population for the overall population and according to sex. The estimated annual average difference with 95% confidence intervals (CIs) was assessed using a generalized linear model with a log-linear link and assuming a Poisson distribution.^10^ The estimated annual percentage change (APC) was expressed as estimated APC = (Exp [estimated annual average difference] −1) × 100. Data preparation and the generalized linear modeling were conducted using SAS 9.4 software (SAS Institute Inc). An age-period-cohort model was used to estimate the net drift, local drifts, and cohort rate ratio in the incidence rate of maintenance dialysis using a web-based tool developed by the National Cancer Institute.^11^ The net drift quantified the overall log-linear trend of the sum of calendar time plus birth cohort. The concept was equivalent to the estimated APC in the ASR. Local drifts evaluated the homogeneity of the age effect on the estimated APC by Wald tests. Cohort rate ratios were compared with those of a a given birth cohort with the mid-1967 reference cohort. We conducted a sensitivity analysis by applying a generalized linear mixed model with a spline function to verify our main results. The knot of the trend change of the ASR in the model was set in 2007. All statistical tests were 2-sided, with *P* values of <0.05 considered statistically significant.

## Results

### Distribution and Trends in the Incidence Rate of Maintenance Dialysis

**Figure 1:**
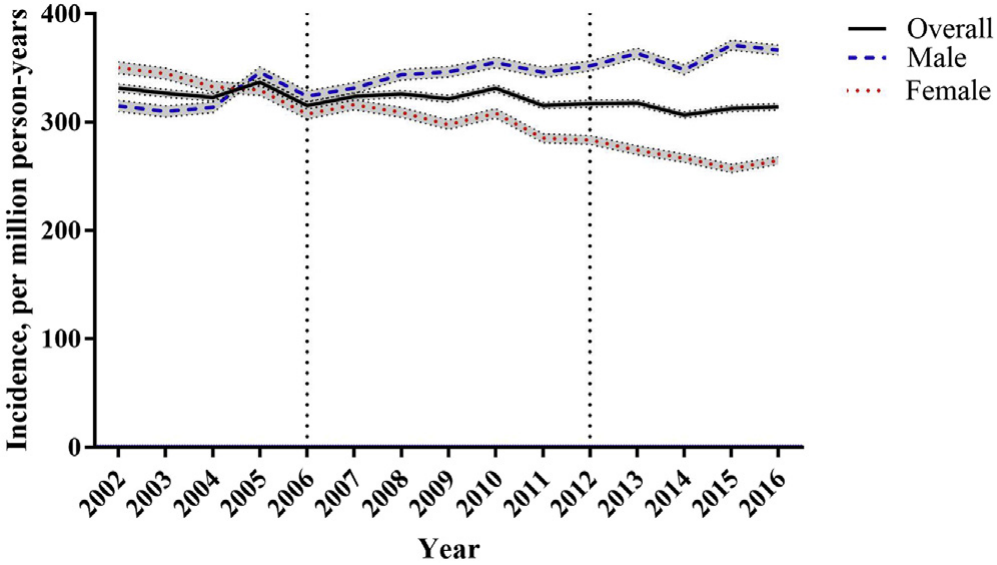
Age-standardized maintenance dialysis incidence rates in Taiwan in 2002-2016. Incidences of maintenance dialysis were collected from the Taiwan National Health Insurance Research Databases, and population information was downloaded from the Population Census Database, Accounting, and Statistics, Executive Yuan, R.O.C. (Taiwan). The age standardizations were implemented through the age distribution of the World Health Organization’s 2000 standard population for overall and different sex groups.

A total of 144,258 incident cases with a follow-up of 346 million person-years were analyzed during 2002-2016. The periodic incidence rate increased from 366.2 per million person-years in 2002-2006 to 465.5 per million person-years in 2012-2016 (Table 1). The incidence rate of women treated by maintenance dialysis was higher than that of men in 2012-2016; however, this relationship was reversed in 2007-2011 and maintained in 2012-2016. The incidence rates increased with age, with the largest absolute difference between 2 adjacent age groups observed in the birth cohort (born in 1943-1947) and age groups of 60-65, 70-74, and 75-79 years in 2002-2006, 2007-2011, and 2011-2016. There was a significant reduction in the APC in women (−2.12; 95% CI, −2.78 to −1.46), and in age groups 45-49 (−2.59; 95% CI, −3.24 to −1.94), 50-54 (−3.02; 95% CI, −3.53 to −2.51), 55-59 (−3.08; 95% CI, −3.49 to −2.67), 60-64 (−1.75; 95% CI, −2.09 to −1.41), and 65-69 (−0.42; 95% CI, −0.72 to −0.12) years. However, age groups over 75 years had a significantly increased annual percentage from 2.21% to 4.24%. After age standardization, the trend in the overall incidence rate was stable, with a slight reduction after 2010 (Fig 1). Women had a higher ASR before 2004, which gradually decreased, whereas men had a lower ASR in 2002, which increased over the 10 years. Finally, a gradual increase in sex differences in the age-standardized incidence rate was observed in recent years. The increased overall incidence trend in men was mainly because of those aged over 65 years, whereas women aged 40-60 years reduced the overall incidence in the female cohort (Table S2 and S3).

**Table 1:** The Period Incidence Rates of Maintenance Dialysis and the Average Annual Percentage Change in the Overall, Sex-Specific, and Age-Specific Groups

	2002-2006			2007-2011			2012-2016			All Observed Years
	Number of Cases	Person-Year of Observation	Rate	Number of Cases	Person-Year of Observation	Rate	Number of Cases	Person-Year of Observation	Rate	Estimated APC (95% CI)
Overall	41,555	113,461,358	366.2	48,167	115,502,198	417.0	54,536	117,154,982	465.5	−0.41 (−1.06 to 0.24)
Sex										
Male	20,403	57,696,203	353.6	25,016	58,152,751	430.2	29,788	58,487,281	509.3	1.15 (0.51 to 1.79)
Female	21,152	55,765,155	379.3	23,151	57,349,447	403.7	24,748	58,667,701	421.8	−2.12 (−2.78 to −1.46)
Age group, y										
0-4	8	6,141,968	1.3	5	5,002,034	1.0	4	5,091,278	0.8	−6.11 (−16.53 to 5.61)
5-9	18	7,643,535	2.4	16	6,202,267	2.6	10	5,073,843	2.0	−1.11 (−8.47 to 6.84)
10-14	45	8,086,771	5.6	37	7,635,666	4.8	25	6,200,118	4.0	−3.72 (−8.78 to 1.61)
15-19	100	8,118,761	12.3	100	8,064,996	12.4	93	7,624,889	12.2	−0.96 (−4.21 to 2.41)
20-24	242	9,535,198	25.4	195	8,098,566	24.1	183	8,043,116	22.8	−1.57 (−3.90 to 0.82)
25-29	496	9,717,501	51.0	428	9,627,961	44.5	356	8,120,627	43.8	−1.29 (−2.97 to 0.43)
30-34	667	9,082,852	73.4	779	9,817,709	79.3	685	9,704,161	70.6	−0.36 (−1.70 to 1.01)
35-39	1,125	9,403,811	119.6	1,032	9,110,280	113.3	1,181	9,834,943	120.1	0.15 (−0.93 to 1.24)
40-44	1,962	9,594,354	204.5	1,642	9,348,147	175.6	1,714	9,062,320	189.1	−0.79 (−1.63 to 0.05)
45-49	3,172	8,875,583	357.4	2,763	9,481,612	291.4	2,588	9,235,492	280.2	−2.59 (−3.24 to −1.94)
50-54	4,482	7,607,509	589.2	4,263	8,727,916	488.4	4,067	9,317,043	436.5	−3.02 (−3.53 to −2.51)
55-59	4,511	4,911,794	918.4	5,644	7,429,871	759.6	5,718	8,519,832	671.1	−3.08 (−3.49 to −2.67)
60-64	4,810	3,968,379	1,212.1	5,710	4,736,071	1,205.6	7,244	7,179,388	1,009.0	−1.75 (−2.09 to −1.41)
65-69	5,574	3,489,538	1,597.3	5,708	3,736,845	1,527.5	6,909	4,500,759	1,535.1	−0.42 (−0.72 to −0.12)
70-74	5,516	2,944,829	1,873.1	6,384	3,152,900	2,024.8	6,473	3,416,598	1,894.6	0.09 (−0.18 to 0.35)
75-79	4,698	2,299,922	2,042.7	6,004	2,482,680	2,418.4	6,903	2,713,711	2,543.7	2.21 (1.96 to 2.45)
80-84	2,709	1,289,302	2,101.1	4,633	1,729,002	2,679.6	5,760	1,914,235	3,009.0	3.57 (3.33 to 3.81)
85+	1,420	749,751	1,894.0	2,824	1,117,675	2,526.7	4,623	1,602,629	2,884.6	4.24 (3.99 to 4.49)

*Note:* Incidence rate expressed per 1,000,000 person-years. We estimated the annual average difference with 95% CIs in the incidence rate of maintenance dialysis in each age group in 2002-2016 by a generalized linear model with a log-linear link assuming a Poisson distribution. Then, we calculated the estimated annual percent change as estimated APC = (Exp [estimated annual average difference] – 1) × 100.

Abbreviations: APC, Annual percent change; CI, confidence interval.

### Distribution and Trends in Prevalence Proportion

There was a 58.3% increase in the periodic prevalence from 2,031.6 per million people in 2002-2006 to 3,216.0 per million people in 2012-2016 observed in the population receiving dialysis (Table 2). The estimated annual percentage increased by 4.61% in the overall group in the observed year, and the estimated annual prevalence increase was more obvious in 2002-2006 and larger in men than in women. In 2002-2006, the estimated APCs significantly increased, particularly in those aged over 40 years (rank, 1.85%-9.78%), whereas there was a much smaller increase in the more recent period (rank, 0.62%-3.83%), even significantly conversed in some age groups (50-54, 55-59, and 60-64 years). In men, there was a significant increase in estimated APCs (1.61%-7.02%) in those aged greater than 40 years (Table S4); however, an increase in estimated APCs (0.59%-6.49%) was only observed in women aged greater than 60 years. Notably, the age range from 40-59 years in the female population represented a 2.61% (95% CI, −2.84 to −2.37) significant reduction in estimated APCs (Table S5).

**Table 2:** The Period Prevalence Proportions of Maintenance Dialysis and the Average Annual Percentage Change in the Overall, Sex-Specific, and Age-Specific Groups

	2002-2006			2007-2011			2012-2016			All Observed Years
	Mean	SD	Estimated APC (95% CI)	Mean	SD	Estimated APC (95% CI)	Mean	SD	Estimated APC (95% CI)	Estimated APC (95% CI)
Overall	2,031.6	219.6	7.09 (5.62 to 8.58)	2,691.3	200.8	4.83 (3.58 to 6.09)	3,216.0	139.8	2.79 (1.67 to 3.92)	4.61 (4.37-4.85)
Sex										
Male	1,848.1	214.2	7.62 (6.07 to 9.19)	2,558.9	234.5	5.97 (4.68 to 7.28)	3,234.8	198.6	3.96 (2.83 to 5.10)	5.64 (5.40-5.90)
Female	2,221.4	224.5	6.60 (5.21 to 8.02)	2,825.8	165.8	3.77 (2.57 to 4.99)	3,197.5	81.4	1.62 (0.51 to 2.74)	3.64 (3.41-3.87)
Age group, y										
0-4	2.6	0.7	1.94 (−30.6 to 49.74)	3.0	1.1	−19.11 (−44.10 to 17.05)	2.0	1.3	−15.38 (−45.84 to 32.21)	−3.35 (−10.26 to 4.09)
5-9	7.2	1.7	−5.67 (−25.17 to 18.90)	10.9	2.0	6.67 (−11.66 to 28.81)	8.1	1.4	5.46 (−15.21 to 31.17)	1.22 (−2.72 to 5.32)
10-14	25.9	2.1	−1.80 (−13.07 to 10.93)	17.8	2.7	3.02 (−11.05 to 19.31)	20.7	1.1	1.66 (−11.29 to 16.49)	−2.06 (−4.51 to 0.45)
15-19	70.8	7.8	−3.95 (−10.77 to 3.40)	65.4	7.6	−5.48 (−12.47 to 2.07)	55.2	3.0	−0.07 (−8.07 to 8.62)	−2.53 (−3.95 to −1.08)
20-24	145.5	6.2	1.71 (−3.39 to 7.07)	159.4	6.3	−2.33 (−7.01 to 2.58)	147.1	8.1	−2.91 (−7.75 to 2.18)	−0.04 (−0.99 to 0.92)
25-29	336.8	8.0	−0.92 (−4.21 to 2.49)	308.5	6.7	−1.12 (−4.55 to 2.43)	311.5	7.3	0.58 (−2.89 to 4.17)	−3.42 (−4.08 to −2.76)
30-34	585.5	16.5	1.71 (−0.86 to 4.35)	610.8	12.6	−0.99 (−3.44 to 1.53)	563.6	18.0	−1.86 (−4.39 to 0.73)	−0.37 (−0.85 to 0.11)
35-39	960.3	12.4	0.56 (−1.43 to 2.59)	978.6	42.6	2.58 (0.57 to 4.63)	1,023.7	29.9	−1.51 (−3.4 to 0.42)	0.63 (0.25 to 1.00)
40-44	1,533.1	53.5	1.91 (0.31 to 3.54)	1,544.9	19.8	−0.76 (−2.31 to 0.82)	1,587.9	79.6	3.14 (1.55 to 4.76)	0.47 (0.17 to 0.77)
45-49	2,480.5	74.6	1.74 (0.48 to 3.01)	2,486.7	19.1	−0.24 (−1.47 to 1.01)	2,413.2	34.9	−0.73 (−1.98 to 0.53)	−0.22 (−0.45 to 0.02)
50-54	3,823.7	121.2	1.85 (0.84 to 2.88)	3,966.3	36.9	−0.41 (−1.39 to 0.57)	3,760.3	100.0	−1.66 (−2.65 to −0.66)	−0.16 (−0.34 to 0.03)
55-59	5,608.0	449.7	4.80 (3.93 to 5.67)	5,935.9	93.2	−0.77 (−1.57 to 0.03)	5,710.5	89.4	−0.97 (−1.78 to −0.15)	0.26 (0.10 to 0.41)
60-64	6,674.8	618.1	5.88 (5.08 to 6.69)	8,454.1	380.9	2.03 (1.34 to 2.72)	8,066.0	309.9	−2.29 (−2.97 to −1.62)	−0.38 (−0.51 to −0.24)
65-69	8,283.0	570.4	4.41 (3.70 to 5.12)	9,703.1	503.6	3.18 (2.53 to 3.83)	11,187.1	358.1	0.93 (0.34 to 1.52)	3.00 (2.88 to 3.12)
70-74	8,925.0	1,130.9	8.36 (7.64 to 9.07)	11,670.5	532.6	2.87 (2.28 to 3.46)	12,430.1	278.8	0.62 (0.07 to 1.19)	0.35 (0.24 to 0.47)
75-79	8,688.3	914.7	6.88 (6.17 to 7.59)	12,147.5	1221.4	6.52 (5.92 to 7.12)	14,252.2	229.1	1.02 (0.50 to 1.55)	4.86 (4.74 to 4.97)
80-84	8,216.3	950.7	7.60 (6.87 to 8.35)	11,604.4	1,046.0	5.87 (5.26 to 6.48)	14,632.6	873.9	3.83 (3.30 to 4.37)	3.07 (2.95 to 3.18)
85+	6,083.8	895.0	9.78 (8.90 to 10.66)	9,473.9	1,203.6	8.18 (7.49 to 8.87)	11,972.0	636.4	3.40 (2.81 to 3.99)	3.97 (3.83 to 4.10)

*Note:* Prevalence expressed per 1,000,000 population. We estimated the annual average difference with 95% CIs in the prevalence proportions of maintenance dialysis in each age group in 2002-2006, 2007-2011, and 2012-2016 by a generalized linear model with a log-linear link assuming a Poisson distribution. Then, we calculated the estimated annual percent change as estimated APC = (Exp [estimated annual average difference] − 1) × 100.

Abbreviations: APC, Annual percent change; CI, confidence interval; SD, standard deviation.

### Trends in Average Dialysis Duration

In general, the average dialysis duration significantly increased from 5.5 (95% CI, 5.3-5.8) in 2002-2006 to 6.9 (95% CI, 6.7-7.2) in 2012-2016 (Table 3), with women having a significantly longer dialysis duration than men. The longest average dialysis duration (≥ 8 years) was observed in those aged 30-59 years. For patients aged greater than 85 years, there was an increase in the average dialysis duration from 3.2 (95% CI, 3.1-3.3) in 2002-2006 to 4.2 (95% CI, 4.1-4.2) in 2012-2016. Similar patterns of estimated APCs across different age groups were observed after stratifying by sex (Tables S6 and S7).

**Table 3:** The Period Average Duration of Maintenance Dialysis in the Overall, Sex-Specific, and Age-Specific Groups

	2002-2006			2007-2011			2012-2016		
	P	I	P/I (95% CI)	P	I	P/I (95% CI)	P	I	P/I (95% CI)
Overall	2,031.6	366.2	5.5 (5.3-5.8)	2,691.30	417.0	6.5 (6.2-6.7)	3,216	465.5	6.9 (6.7-7.2)
Sex									
Male	1,848.1	353.6	5.2 (5.0-5.5)	2,558.90	430.2	5.9 (5.7-6.2)	3,234.80	509.3	6.4 (6.1-6.6)
Female	2,221.4	379.3	5.9 (5.6-6.1)	2,825.80	403.7	7.0 (6.7-7.3)	3,197.50	421.8	7.6 (7.3-7.8)
Age group, y									
0-4	2.6	1.3	2.0 (0.3-6.1)	3.0	1	3.0 (0.6-8.8)	2	0.8	2.5 (0.3-9.2)
5-9	7.2	2.4	3.1 (1.2-6.1)	10.9	2.6	4.2 (2.0-7.4)	8.1	2.0	4.1 (1.8-8.0)
10-14	25.9	5.6	4.6 (3.0-6.7)	17.8	4.8	3.7 (2.1-5.7)	20.7	4.0	5.1 (3.1-7.8)
15-19	70.8	12.3	5.7 (4.5-7.2)	65.4	12.4	5.3 (4.0-6.7)	55.2	12.2	4.5 (3.4-5.9)
20-24	145.5	25.4	5.7 (4.8-6.7)	159.4	24.1	6.6 (5.6-7.7)	147.1	22.8	6.5 (5.5-7.6)
25-29	336.8	51.0	6.6 (5.9-7.3)	308.5	44.5	6.9 (6.2-7.8)	311.5	43.8	7.1 (6.3-7.9)
30-34	585.5	73.4	8.0 (7.3-8.6)	610.8	79.3	7.7 (7.1-8.3)	563.6	70.6	8.0 (7.3-8.7)
35-39	960.3	119.6	8.0 (7.5-8.5)	978.6	113.3	8.6 (8.1-9.2)	1,023.70	120.1	8.5 (8.0-9.1)
40-44	1,533.1	204.5	7.5 (7.1-7.9)	1,544.90	175.6	8.8 (8.4-9.2)	1,587.90	189.1	8.4 (8.0-8.8)
45-49	2,480.5	357.4	6.9 (6.7-7.2)	2,486.70	291.4	8.5 (8.2-8.9)	2,413.20	280.2	8.6 (8.3-9.0)
50-54	3,823.7	589.2	6.5 (6.3-6.7)	3,966.30	488.4	8.1 (7.9-8.4)	3,760.30	436.5	8.6 (8.3-8.9)
55-59	5,608	918.4	6.1 (5.9-6.3)	5,935.90	759.6	7.8 (7.6-8.0)	5,710.50	671.1	8.5 (8.3-8.7)
60-64	6,674.8	1,212.1	5.5 (5.4-5.6)	8,454.10	1,205.60	7.0 (6.9-7.2)	8,066.00	1,009.0	8.0 (7.8-8.2)
65-69	8,283	1,597.3	5.2 (5.1-5.3)	9,703.10	1,527.50	6.4 (6.2-6.5)	11,187.10	1,535.1	7.3 (7.2-7.4)
70-74	8,925	1,873.1	4.8 (4.7-4.9)	11,670.50	2,024.80	5.8 (5.7-5.9)	12,430.10	1,894.6	6.6 (6.4-6.7)
75-79	8,688.3	2,042.7	4.3 (4.2-4.3)	12,147.50	2,418.40	5.0 (4.9-5.1)	14,252.20	2,543.7	5.6 (5.5-5.7)
80-84	8,216.3	2,101.1	3.9 (3.8-4.0)	11,604.40	2,679.60	4.3 (4.3-4.4)	14,632.60	3,009.0	4.9 (4.8-4.9)
85+	6,083.8	1,894.0	3.2 (3.1-3.3)	9,473.90	2,526.70	3.7 (3.7-3.8)	11,972.00	2,884.6	4.2 (4.1-4.2)

*Note:* The 95% CIs on the P/I were estimated from the 95% CI on P, assuming a Poisson distribution of prevalence. The variance of log P, V(log P) = 1/Number of maintenance dialysis.

Abbreviations: CI, Confidence interval; I, incidence; P, prevalence.

### Trends in Mortality Rate in the Dialysis Population

Annual mortality rates in the population receiving dialysis were consistently stable (range, 10.8-11.6%), with a 1.8 (95% CI, −1.8 to 5.6) estimated APC in the 3 periodic periods (Table 4). Men showed a slightly higher mortality rate than women. A U-shape relationship was found between age and mortality rate in each period. The minimum mortality rate was in the age group 20-24 years (range, 1.2%-1.6%), and nearly 40% of patients with age greater than or equal to 85 years died every year under the insurance system. Notably, the old age groups 70-79 and 80-84 years exhibited an obvious improvement in their mortality rate by 5.0 (95% CI, 2.3-7.7) and 2.3 (95% CI, 0.1-4.5) reduction, respectively. Except for the unreliable estimation in APCs causing few deaths in the population under teenage ages, the male population had significantly lower estimated APCs (range, −3.22% to −4.55%) in those aged between 55 and 79 years, whereas a significant reduction in the estimated APCs (range: −2.15% to −5.14%) was observed in the female population aged greater than 55 years (Tables S8 and S9).

**Table 4:** The Period Mortality Rates of Maintenance Dialysis and the Average Annual Percentage Change in the Overall, Sex-Specific, and Age-Specific Groups

	2002-2006			2007-2011			2012-2016			All observed years
	Number of Cases	Patient-Year of Observation	Rate	Number of Cases	Patient-Year of Observation	Rate	Number of Cases	Patient-Year of Observation	Rate	Estimated APC (95% CI)
Overall	24,676	221,450.5	11.1	32,685	303,207	10.8	42,850	370,973	11.6	1.8 (−1.8 to 5.6)
Sex										
Male	12,378	102,343.5	12.1	16,406	144,520	11.4	22,120	185,373	11.9	−2.1 (−5.5 to 1.5)
Female	12,298	119,107	10.3	16,279	158,687	10.3	20,730	185,601	11.2	0.7 (−2.9 to 4.4)
Age group, y										
0-4	3	16	18.8	1	16.5	6.1	1	10	10.0	−15.4 (−19.2 to −11.4)
5-9	6	58	10.4	5	67	7.5	1	42	2.4	−15.6 (−19.9 to −11.2)
10-14	6	197	3.0	11	141	7.8	5	132	3.8	−2.5 (−7.7 to 3.0)
15-19	14	557	2.5	14	518	2.7	7	427	1.6	−5.4 (−12.6 to 2.3)
20-24	21	1,328	1.6	17	1,268	1.3	14	1,188	1.2	−2.8 (−12.8 to 8.4)
25-29	62	3,111	2.0	50	2,983	1.7	35	2,527	1.4	−7.1 (−16.9 to 3.9)
30-34	105	5,100	2.1	128	5,879	2.2	121	5,596	2.2	1.9 (−5.9 to 10.4)
35-39	194	8,669	2.2	210	8,684	2.4	255	9,979	2.6	1.1 (−6.3 to 9.0)
40-44	409	13,970	2.9	382	14,210	2.7	425	14,168	3.0	−0.3 (−7.2 to 7.1)
45-49	815	20,769	3.9	778	23,066	3.4	791	22,281	3.6	1.2 (−5.1 to 7.8)
50-54	1,484	27,001	5.5	1,508	33,647	4.5	1,572	34,942	4.5	−0.7 (−6.2 to 5.2)
55-59	2,018	25,072	8.0	2,674	42,122	6.3	2,955	48,191	6.1	−1.6 (−6.3 to 3.2)
60-64	2,561	24,858	10.3	3,417	37,458	9.1	4,654	56,500	8.2	−2.4 (−6.1 to 1.5)
65-69	3,569	26,960	13.2	3,901	35,229	11.1	5,116	47,928	10.7	−2.2 (−5.5 to 1.2)
70-74	4,343	24,441	17.8	5,141	35,440	14.5	5,854	42,312	13.8	−1.2 (−4.2 to 1.9)
75-79	4,179	18,447	22.7	5,565	29,197	19.1	7,013	38,013	18.4	−5.0 (−7.7 to −2.3)
80-84	2,992	9,781	30.6	4,941	19,313	25.6	6,822	27,720	24.6	−2.3 (−4.5 to −0.1)
85+	1,894	4,182	45.3	3,942	10,264	38.4	7,209	18,995	38.0	0.2 (−1.8 to 2.2)

*Note:* Mortality rate expressed per 100 person-years.

We estimated the annual average difference with 95% CIs in the mortality rate of maintenance dialysis in each age group in 2002-2016 by a generalized linear model with a log-linear link assuming a Poisson distribution. Then, we calculated the estimated annual percent change as estimated APC = (Exp [estimated annual average difference] − 1)✕100.

Abbreviations: APC, Annual percent change; CI, confidence interval.

### Age-Period-Cohort Effect on the Periodic Incidence

**Figure 2:**
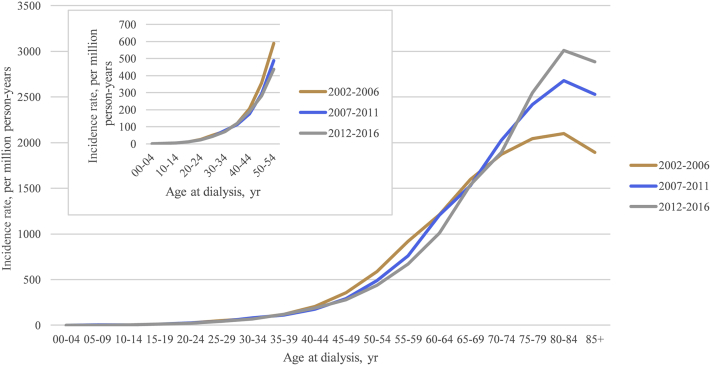
The age-specific incidence rate of maintenance dialysis in Taiwan by year of dialysis (2002-2006, 2007-2011, and 2012-2016). Incidences of maintenance dialysis were collected from the Taiwan National Health Insurance Research Databases, and population information was downloaded from the Population Census Database, Accounting, and Statistics, Executive Yuan, R.O.C. (Taiwan).

**Figure 3:**
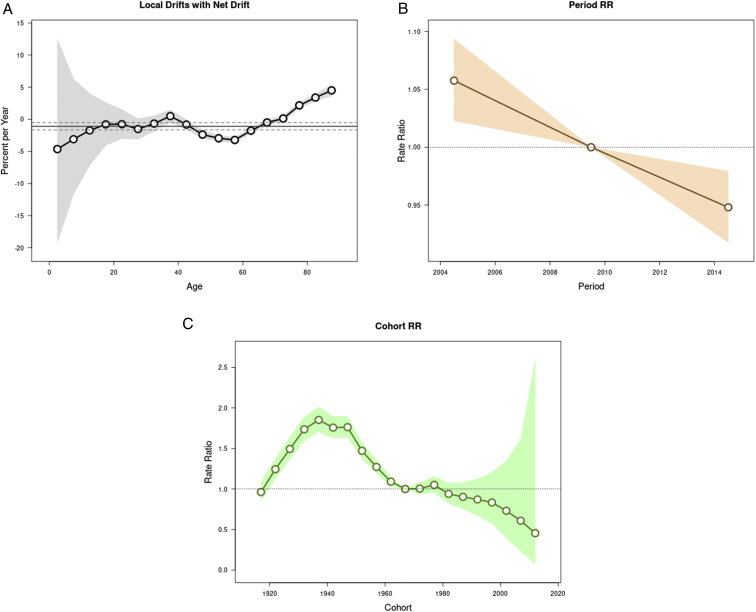
The effects of age, period, and cohort on the incidence rate of maintenance dialysis in Taiwan. (A) Age-specific net annual percentage change (local drift) for the incidence rate of maintenance dialysis. (B) Period rate ratio (RR) (horizontal yellow line) and 95% confidence intervals (yellow shading) for the incidence rate of maintenance dialysis. (C) Cohort rate ratios (RR) (horizontal green line) and 95% confidence intervals (green shading) for incidence of maintenance dialysis. The vertical lines in (B) indicate a rate ratio of 1 (no difference between the selected reference period 2007-2011). The vertical lines indicate a rate ratio of 1 (no difference between the selected reference birth cohort 1965-1969). The model was fitted using the National Cancer Institute’s Age Period Cohort web tool, and the figure was directly produced from the web (analysistools.cancer.gov/apc/).

**Figure 4:**
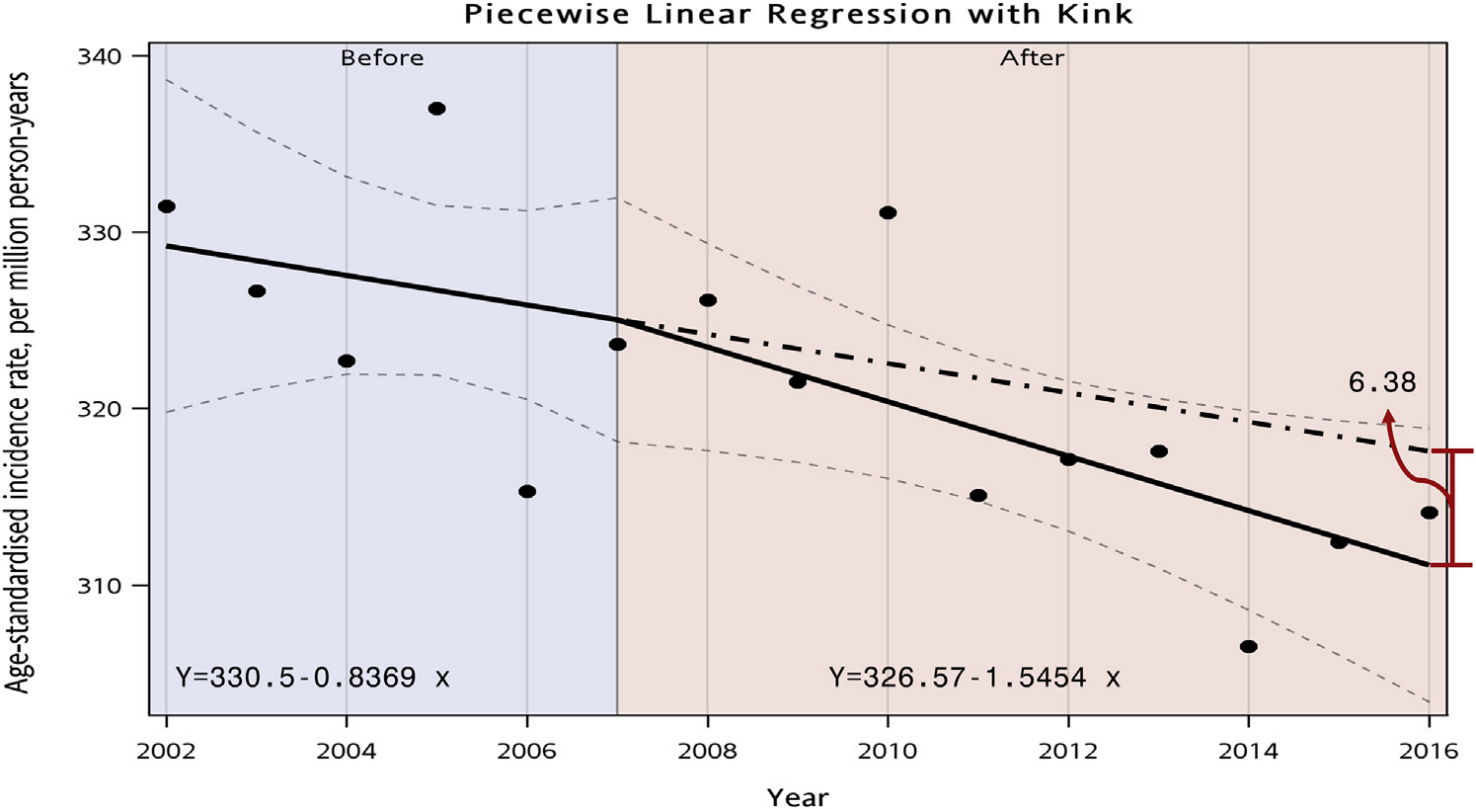
Trends of age-standardized incidence rate of maintenance dialysis in 2002-2007 and 2007-2016. The model was developed by a generalized linear mixed model with a spline function by setting knot in 2007. The regression equation is Y=330.5-0.83369 X for the first phase (2002-2007) and Y=326.57-1.5454 X for the second phase (2007-2016), where X is a calendar year and Y is the age-standardized incidence rate of maintenance dialysis in 1 year. The dashed line represents the extrapolation of the slope developed in 2002-2007 to 2016.

The age-specific incidence rate by the 3 periods (2002-2006, 2007-2011, and 2012-2016) demonstrated that the recent period 2012-2016 had the lowest incidence rates for most of the age groups except for age group of greater than 65 years (Fig 2). The relationship between age and APC after considering the influences of period represents a J shape. There was an insignificant APC in incidence rates in those aged lesser than 40 years but a significant reduction in APC from 0.8 among the population aged 40-44 years to 0.5 among the population aged 65-69 years. After the age of 75 years, the APC increased from 2.2 to 4.5 per year (Fig 3A). The maintenance dialysis incidence was significantly reduced over time, with an overall net drift of −1.09% (95% CI, −1.65 to −0.52) per year and a significant linear reduction in period rate ratio from 1.06 (95% CI, 1.02-1.09) in the years 2002-2006 to 0.95 (95% CI, 0.92-0.98) in the years 2012-2016 when taking the years 2007-2011 as reference (Fig 3B). Moreover, a noticeable inverted V-shape with a tableland nearly double ratio in 1930-1934, 1935-1939, 1940-1944, and 1945-1950 birth cohorts (range of ratio, 1.74-1.85) was observed when treating the 1965-1969 birth cohort as the reference group. For cohorts that were younger or older than the range of cohort, the ratio steadily decreased. Despite nonsignificance, the incidence rate tended to be lower in the birth cohorts younger than 1980 than the reference group (Fig 3C). The sensitivity analysis results reconfirmed our main findings. Using spline functions to model ASR change before and after 2007, we found that the slope in the more recent period (−1.54) was steeper than that in the preceding one (−0.84). Without the CKD care policies, an increase of 6.38 per million person-years of ASR, equivalent to 150 new cases from actual observation, could be anticipated in 2016 (Fig 4).

## Discussion

Current findings demonstrated that the temporal trend of the incidence rate of maintenance dialysis in Taiwan substantially declined by almost 10% annually before and after the national incentivized CKD care policies were implemented. We also quantified the age and cohort effects on incidence rates by analyzing the age-period-cohort model. In addition, the different increases in age- and sex-specific incidence rates, together with the stabilization of mortality rates in maintenance dialysis, might explain the heterogeneous rising trend in prevalence between age groups.

The efficacy of the CKD care programs has been largely reported in recent years.^12^, ^13^, ^14^, ^15^ Similar to these individual-level observations, our findings reflect that the universal CKD care programs could decline the long-term trend of dialysis incidence. Although the fundamental mechanisms of the results are yet to be clarified, recent evidence suggests that more appropriate medication prescriptions,^5^^,^^16^ education and dietary interventions,^17^^,^^18^ complication management,^19^ dialysis preparation,^20^ as well as their interactions may play essential roles in prolonging kidney disease progression and dialysis initiation. In addition, our results, together with previous reports,^5^^,^^12^^,^^21^ suggested that the care programs could significantly reduce cardiovascular disease or infectious disease-related mortality, particularly in the first year of dialysis, through improving the electrolyte imbalance correction and arteriovenous fistula preparation.^12^ It is true that the Taiwan government must pay additional fees for patient education, dietician consultations, and laboratory examinations when it performs the 2 CKD care programs; however, overall, the cost is lower than that of conventional care when the programs prevent patients from starting dialysis or repeated hospitalizations.^12^^,^^13^ As a result, it is highly encouraged to apply the concept of the care programs to other developed countries and evaluate their effectiveness and costs.

Age has been generally applied to explain the differences in incidence rates of maintenance dialysis between countries when performing comparisons.^22^ The standardization of incidence rates by the age distribution of a selected standard population is a common approach to compensate for the different effects of age contributing to incidence rates between countries. However, age-group heterogeneity effects may exist, causing oversimplified results and biased interpretation. The current study applied an age-period-cohort approach to clarify the above conditions and demonstrate that different birth cohorts substantially affect the occurrence of kidney failure. In our analyses, the birth cohort 1943-1947 (during the period of World War II) had the highest risk of kidney failure compared with the other birth cohorts; hence, health policymakers should consider the influence of poor environmental conditions caused by war at birth on the risk of kidney disease.^23^ Although the care program might suggest conservative nondialysis management rather than KRT to some severely ill elderly patients, hospice care in Taiwan would not be initiated unless a life-threatening condition occurs.^24^^,^^25^ It may explain why the care program does not significantly impact the incidence rates of elders.

Trends in the maintenance dialysis incidence rates during our study periods (2006-2016) significantly decreased after controlling for age and cohort effects. The period effect may be explained by the efficacy of the universal incentive CKD care programs associated with reducing the risk of kidney failure that have been previously demonstrated in several individual studies.^12^, ^13^, ^14^, ^15^ The programs may improve patient kidney function progression by regularly evaluating proteinuria and kidney function, providing appropriate management, thereby reducing the incidence rates. However, the identified trend is inconsistent with the descriptions in the annual report of the US Renal Data System. The US Renal Data System calculated each country’s crude (unadjusted) incidence rate by collecting the accumulated data from different national kidney registries and census data. This approach is convenient for understanding but may neglect the diversities of the completeness of the kidney registry systems across time and age compositions between countries. Therefore, more studies exploring those effects on maintenance dialysis incidence rates between countries or regions are warranted.

The annual standardized maintenance dialysis incidence rate represented different trends for sex in the current study, possibly caused by the complex interactions of social, biological, and behavioral factors. Social, cultural, and health care factors might cause sex discrepancy in treatment^26^; however, the last factor is less likely in our study under the universal health care insurance system. Men are more likely than women to have poor life habits and work in dangerous conditions, which may lead to a larger number of men progressing faster to kidney failure.^27^ Further research applying more comprehensive data to clarify the role of sex in the natural history of CKD is needed.

The prevalence of maintenance dialysis is influenced by a dynamic balance of inflows (incidence rates) and outflows (mortality rates). The increase in estimated APC of prevalence proportion but the decrease in net incidence rate across the observed period reflects that the survival of the population receiving dialysis is substantially improved by health care, which is also reflected by the longer average dialysis duration in recent periods. Furthermore, the remarkably high incidence and mortality rates of maintenance dialysis in the age group greater than or equal to 75 years prompts a discussion of the effect of social values on health care resources sustainable utilization and the value of extending life by dialysis care.^28^

Several strengths of the present study should be emphasized. First, the single universal health care system with strict regulations that covers all medical needs reduces the uncertainty of data quality from different sources. Second, a long follow-up time allows the effects of the universal CKD programs, age, and cohort on maintenance dialysis incidence to be determined. Nonetheless, there were several limitations. The policy affecting medical decisions in dialysis therapy may, to some degree, influence our results. For example, the number of patients receiving kidney transplantation and conservative care before dialysis may change by the reimbursement policy over time. It might cause changes in our estimations (Table S10). However, there would only be a few cases because of organ shortage and the Eastern culture of death denial, which makes the influences on our estimations less likely.^29^ Third, patients may go overseas to seek appropriate treatment in some countries; however, this is less likely in Taiwan because of the relatively high-quality and low-cost health care compared with adjacent countries. Finally, because of the special and comprehensive features of our health care system, generalizing our results to different health care systems should be performed with caution.

In conclusion, the implementation of universal incentive CKD care programs in Taiwan has been significantly associated with declining the long-term trend of maintenance dialysis incidence and a significant annual increase in the prevalence proportion. On the basis of our findings, devoting governmental resources to CKD care and prevention is advocated.
