# Supplementary material for: CKD Care Programs and Incident Kidney Failure: A Study of a National Disease Management Program in Taiwan
Source: Kidney Med. 2022 May 21;4(7):100485. doi: 10.1016/j.xkme.2022.100485 (PMC9257411; doi:10.1016/j.xkme.2022.100485)
Supplement: Supplementary File (PDF) — Tables S1-S10. [file mmc1.pdf]

Table S1. The reimbursement codes for clinical treatment are provided by the Taiwan National Health Insurance

**Clinical treatment**	**Database[#]**	**Corresponding reimbursement codes[$]**
Hemodialysis treatment per patient visit	Heath04	58001C, 58019C, 58020C, 58021C, 58022C, 58023C, 58024C, 58025C, 58027C, 58029C
Peritoneal dialysis care	Health04	58002C, 58009A, 58009B, 58010A, 58010B, 58011A, 58011AB, 58011B, 58011C, 58012A, 58012B, 58017B, 58017C, 58026C, 58028C
Surgery for kidney transplantation	Health05	76020A, 76020B, 97416K, 97417A, 97418B

[#]Data sources were from Health04 (Details of ambulatory care orders), and Health05 (Details of hospitalization admission care orders). Detailed information can be accessed at https://dep.mohw.gov.tw/dos/lp-3147-113.html.

[$]Information can be accessed at http://www.nhi.gov.tw/query/query2.aspx?menu=20&menu_id=712&WD_ID=830.

Table S2. The period incidence rates of maintenance dialysis and the average annual percentage change in the age-specific male population

	2002–2006			2007–2011			2012–2016			All observed years
	Number of cases	Person-year of observation	Rate	Number of cases	Person-year of observation	Rate	Number of cases	Person-year of observation	Rate	Estimated APC (95% CI)
Age group, y										
00–04	5	3,208,982	1.6	0	2,612,211	0.0	3	2,640,510	1.1	-2.83 (-14.1, 9.92)
05–09	12	3,979,707	3.0	10	3,239,789	3.1	8	2,649,545	3.0	0.13 (-6.45, 7.16)
10–14	25	4,216,836	5.9	19	3,974,331	4.8	15	3,238,884	4.6	-3.09 (-8.04, 2.11)
15–19	60	4,203,820	14.3	55	4,200,063	13.1	55	3,965,928	13.9	-1.24 (-4.33, 1.95)
20–24	136	4,885,956	27.8	97	4,179,071	23.2	98	4,181,494	23.4	-2.19 (-4.48, 0.16)
25–29	249	4,940,529	50.4	226	4,869,653	46.4	193	4,164,266	46.3	-0.87 (-2.54, 0.83)
30–34	371	4,587,744	80.9	415	4,909,757	84.5	368	4,842,936	76.0	-0.35 (-1.64, 0.97)
35–39	645	4,761,424	135.5	628	4,542,828	138.2	704	4,868,187	144.6	1.02 (0.02, 2.03)
40–44	995	4,852,064	205.1	951	4,690,765	202.7	1,069	4,480,871	238.6	1.73 (0.92, 2.54)
45–49	1,634	4,462,376	366.2	1,660	4,747,671	349.6	1,735	4,592,742	377.8	0.07 (-0.54, 0.68)
50–54	2,378	3,802,915	625.3	2,520	4,336,499	581.1	2,629	4,614,761	569.7	-1.29 (-1.76, -0.81)
55–59	2,427	2,435,117	996.7	3,366	3,660,805	919.5	3,592	4,178,794	859.6	-1.54 (-1.92, -1.17)
60–64	2,443	1,931,383	1,264.9	3,164	2,307,714	1,371.1	4,381	3,482,024	1,258.2	0.20 (-0.13, 0.52)
65–69	2,498	1,665,443	1,499.9	2,804	1,779,811	1,575.4	3,744	2,149,434	1,741.9	1.91 (1.61, 2.20)
70–74	2,454	1,498,794	1,637.3	2,939	1,459,913	2,013.1	3,260	1,582,569	2,059.9	2.32 (2.05, 2.6)
75–79	2,180	1,252,363	1,740.7	2,663	1,214,569	2,192.5	3,173	1,205,227	2,632.7	4.07 (3.81, 4.34)
80–84	1,242	665,270	1,866.9	2,206	897,809	2,457.1	2,543	885,047	2,873.3	3.86 (3.61, 4.11)
85+	649	345,480	1,878.5	1,293	529,492	2,442.0	2,218	764,062	2,902.9	4.34 (4.09, 4.59)

Abbreviations: APC, annual percent change; CI, confidence interval.

Incident rate expressed per 1 000 000 person-years.

We estimated the annual average difference with 95% confidence intervals in the incidence rate of maintenance dialysis in each age group in 2002–2016 by a generalized linear model with a log-linear link assuming a Poisson distribution. Then, we calculated the estimated annual percent change as estimated APC=[Exp(estimated annual average difference)-1]×100.

Table S3. The period incidence rates of maintenance dialysis and the average annual percentage change in the age-specific female population

	2002–2006			2007–2011			2012–2016			All observed years
	Number of cases	Person-year of observation	Rate	Number of cases	Person-year of observation	Rate	Number of cases	Person-year of observation	Rate	Estimated APC (95% CI)
Age group, y										
00–04	3	2,932,986	1.0	5	2,389,823	2.1	1	2,450,768	0.4	-8.47 (-18.37, 2.62)
05–09	6	3,663,828	1.6	6	2,962,478	2.0	2	2,424,298	0.8	-3.85 (-12.75, 5.95)
10–14	20	3,869,935	5.2	18	3,661,335	4.9	10	2,961,234	3.4	-5.05 (-10.28, 0.49)
15–19	40	3,914,941	10.2	45	3,864,933	11.6	38	3,658,961	10.4	-0.59 (-4.09, 3.03)
20–24	106	4,649,242	22.8	98	3,919,495	25.0	85	3,861,622	22.0	-0.66 (-3.05, 1.80)
25–29	247	4,776,972	51.7	202	4,758,308	42.5	163	3,956,361	41.2	-2.13 (-3.83, -0.4)
30–34	296	4,495,108	65.8	364	4,907,952	74.2	317	4,861,225	65.2	-0.60 (-200, 0.82)
35–39	480	4,642,387	103.4	404	4,567,452	88.5	477	4,966,756	96.0	-0.69 (-1.87, 0.51)
40–44	967	4,742,290	203.9	691	4,657,382	148.4	645	4,581,449	140.8	-4.04 (-4.92, -3.15)
45–49	1,538	4,413,207	348.5	1,103	4,733,941	233.0	853	4,642,750	183.7	-6.61 (-7.30, -5.90)
50–54	2,104	3,804,594	553.0	1,743	4,391,417	396.9	1,438	4,702,282	305.8	-6.01 (-6.55, -5.46)
55–59	2,084	2,476,677	841.5	2,278	3,769,066	604.4	2,126	4,341,038	489.7	-5.72 (-6.16, -5.29)
60–64	2,367	2,036,996	1,162.0	2,546	2,428,357	1,048.4	2,863	3,697,364	774.3	0.20 (-0.13, 0.52)
65–69	3,076	1,824,095	1,686.3	2,904	1,957,034	1,483.9	3,165	2,351,325	1,346.0	-2.04 (-2.33, -1.75)
70–74	3,062	1,446,035	2,117.5	3,445	1,692,987	2,034.9	3,213	1,834,029	1,751.9	-2.07 (-2.32, -1.81)
75–79	2,518	1,047,559	2,403.7	3,341	1,268,111	2,634.6	3,730	1,508,484	2,472.7	0.37 (0.14, 0.61)
80–84	1,467	624,032	2,350.8	2,427	831,193	2,919.9	3,217	1,029,188	3,125.8	2.66 (2.44, 2.89)

85+	771	404,271	1,907.1	1,531	588,183	2,602.9	2,405	838,567	2,868.0	4.01 (3.77, 4.25)

Abbreviations: APC, annual percent change; CI, confidence interval.

Incident rate expressed per 1 000 000 person-years.

We estimated the annual average difference with 95% confidence intervals in the incidence rate of maintenance dialysis in each age group in 2002–2016 by a generalized linear model with a log-linear link assuming a Poisson distribution. Then, we calculated the estimated annual percent change (APC) as estimated APC=[Exp(estimated annual average difference)-1]×100.

Table S4. The period prevalence proportions of maintenance dialysis and the average annual percentage change in the age-specific male population

	2002–2006			2007–2011			2012–2016			All observed years
	Mean	SD	Estimated APC (95% CI)	Mean	SD	Estimated APC (95% CI)	Mean	SD	Estimated APC (95% CI)	Estimated APC (95% CI)
Age group, y										
00–04	1.3	0.7	20.39 (-31.3, 110.99)	0.4	0.8	-100.00 (-100.00, -100.00)	1.5	1.6	-24.55 (-55.41, 27.67)	0.34 (-10.46, 12.44)
05–09	8.8	2.2	-3.99 (-22.14, 18.38)	10.8	2.7	-3.75 (-20.34, 16.30)	10.7	3.6	23.0 (1.21, 49.47)	2.22 (-1.50, 6.07)
10–14	26.1	3.4	-2.92 (-14.02, 9.61)	19.7	3.0	7.52 (-6.54, 23.70)	19.6	2.7	6.57 (-7.39, 22.64)	-2.33 (-4.76, 0.16)
15–19	82.8	11.8	-6.15 (-12.35, 0.48)	69.8	7.1	-3.86 (-10.75, 3.55)	60.4	4.2	-3.14 (-10.57, 4.90)	-3.27 (-4.62, -1.91)
20–24	169.2	11.0	3.48 (-1.34, 8.53)	175.2	15.5	-5.22 (-9.56, -0.67)	160.7	6.5	-1.20 (-5.91, 3.75)	-0.57 (-1.46, 0.33)
25–29	355.8	10.2	-1.22 (-4.41, 2.08)	339.5	9.0	0.36 (-2.96, 3.79)	330.1	12.9	-1.43 (-4.73, 2.00)	-0.75 (-1.38, -0.12)
30–34	636.6	17.1	1.52 (-0.95, 4.04)	647.8	9.9	0.40 (-2.02, 2.87)	622.2	15.4	-1.15 (-3.57, 1.34)	-0.17 (-0.64, 0.29)
35–39	1,049.0	30.8	1.59 (-0.33, 3.55)	1,092.8	52.0	2.80 (0.89, 4.74)	1,137.3	35.8	-1.47 (-3.27, 0.35)	0.82 (0.47, 1.18)
40–44	1,492.6	99.9	4.27 (2.60, 5.95)	1,707.1	27.0	0.86 (-0.64, 2.39)	1,874.4	123.7	4.15 (2.67, 5.66)	2.37 (2.08, 2.66)
45–49	2,281.0	97.2	2.65 (1.33, 3.99)	2,553.6	82.4	2.04 (0.80, 3.30)	2,808.1	53.6	1.15 (-0.02, 2.34)	2.07 (1.84, 2.31)
50–54	3,567.0	149.7	2.50 (1.44, 3.57)	3,964.6	98.4	1.40 (0.40, 2.40)	4,200.8	43.9	0.54 (-0.41, 1.51)	1.61 (1.42, 1.80)
55–59	5,293.7	549.4	6.51 (5.61, 7.43)	6,065.4	97.7	0.66 (-0.14, 1.46)	6,212.0	94.1	0.84 (0.05, 1.64)	1.67 (1.52, 1.83)
60–64	6,322.0	711.8	7.22 (6.39, 8.06)	8,472.2	533.3	3.54 (2.85, 4.24)	8,711.4	233.4	-1.37 (-2.02, -0.71)	3.06 (2.92, 3.20)
65–69	7,520.3	693.0	5.96 (5.21, 6.73)	9,407.8	722.9	4.87 (4.20, 5.55)	11,625.4	663.9	3.04 (2.45, 3.64)	4.44 (4.31, 4.57)
70–74	7,344.4	1,121.0	10.13 (9.34, 10.94)	10,657.3	782.8	4.74 (4.11, 5.37)	12,353.6	448.0	2.00 (1.43, 2.57)	5.10 (4.98, 5.23)

75–79	7,198.8	638.3	5.76 (4.99, 6.54)	10,378.6	1,390.9	8.85 (8.18, 9.51)	13,501.6	652.2	3.08 (2.53, 3.63)	6.29 (6.16, 6.41)
80–84	7,284.8	812.3	7.28 (6.5, 8.06)	10,039.2	785.8	5.05 (4.40, 5.70)	13,138.8	1,159.4	5.71 (5.14, 6.28)	6.00 (5.88, 6.13)
85+	5,617.3	674.0	7.78 (6.89, 8.68)	8,833.8	1,243.4	8.99 (8.27, 9.71)	11,459.3	570.4	3.15 (2.55, 3.75)	7.02 (6.89, 7.16)

Abbreviations: APC, annual percent change; CI, confidence interval.
Prevalence expressed per 1 000 000 population.
We estimated the annual average difference with 95% confidence intervals in the prevalence proportions of maintenance dialysis in each age group in 2002–2006, 2007–2011, and 2012–2016 by a generalized linear model with a log-linear link assuming a Poisson distribution. Then, we calculated the estimated annual percent change as estimated APC=[Exp(estimated annual average difference)-1]×100.

Table S5. The period prevalence proportions of maintenance dialysis and the average annual percentage change in the age-specific female population

	2002–2006			2007–2011			2012–2016			All observed years
	Mean	SD	Estimated APC (95% CI)	Mean	SD	Estimated APC (95% CI)	Mean	SD	Estimated APC (95% CI)	Estimated APC (95% CI)
Age group, y										
00–04	4.1	0.8	-2.78 (-28.48, 32.17)	5.8	1.5	-14.47 (-34.13, 11.05)	2.5	1.0	-9.97 (-39.51, 34.00)	-4.53 (-9.95, 1.21)
05–09	5.4	1.5	-8.77 (-30.18, 19.2)	11.0	3.3	19.26 (-1.43, 44.3)	5.3	2.9	-25.4 (-43.89, -0.84)	-0.22 (-4.47, 4.22)
10–14	25.6	2.2	-0.48 (-11.96, 12.49)	15.8	3.9	-2.61 (-16.67, 13.83)	21.9	1.6	-2.95 (-15.00, 10.81)	-1.76 (-4.24, 0.79)
15–19	58.0	5.7	-0.56 (-8.33, 7.87)	60.6	9.2	-7.42 (-14.53, 0.29)	49.6	4.3	4.02 (-4.76, 13.6)	-1.53 (-3.06, 0.03)
20–24	120.6	4.4	-0.88 (-6.32, 4.87)	142.7	4.3	1.52 (-3.61, 6.93)	132.3	11.4	-5.13 (-10.12, 0.13)	0.64 (-0.38, 1.67)
25–29	317.1	10.8	-0.56 (-3.96, 2.97)	276.7	14.4	-2.94 (-6.49, 0.75)	292.1	14.3	2.94 (-0.73, 6.74)	-0.77 (-1.45, -0.09)
30–34	533.3	17.0	2.00 (-0.70, 4.78)	573.8	26.2	-2.50 (-5.00, 0.05)	505.2	23.0	-2.77 (-5.41, -0.05)	-0.59 (-1.09, -0.08)
35–39	869.5	14.0	-0.66 (-2.73, 1.45)	865.2	35.1	2.40 (0.26, 4.58)	912.3	25.0	-1.54 (-3.54, 0.50)	0.44 (0.04, 0.83)
40–44	1,574.7	42.8	-0.32 (-1.86, 1.25)	1,381.8	59.8	-2.69 (-4.30, -1.05)	1,308.0	39.6	1.85 (0.12, 3.61)	-1.71 (-2.02, -1.40)
45–49	2,682.3	60.3	0.94 (-0.26, 2.16)	2,420.0	102.7	-2.59 (-3.81, -1.35)	2,022.9	107.4	-3.20 (-4.53, -1.86)	-2.61 (-2.84, -2.37)
50–54	4,080.4	94.1	1.27 (0.30, 2.26)	3,968.3	138.3	-2.16 (-3.12, -1.2)	3,328.3	231.2	-4.30 (-5.32, -3.26)	-1.93 (-2.11, -1.74)
55–59	5,917.3	371.4	3.31 (2.48, 4.14)	5,810.6	209.8	-2.20 (-2.99, -1.40)	5,227.8	251.7	-2.99 (-3.82, -2.16)	-1.14 (-1.30, -0.99)
60–64	7,009.1	530.5	4.77 (3.99, 5.55)	8,437.2	296.6	0.60 (-0.08, 1.28)	7,458.3	400.5	-3.29 (-3.98, -2.59)	0.59 (0.45, 0.72)
65–69	8,981.0	450.2	3.16 (2.49, 3.84)	9,971.0	315.6	1.76 (1.13, 2.39)	10,786.4	333.2	-1.11 (-1.70, -0.52)	1.76 (1.64, 1.88)
70–74	10,567.8	992.1	6.10 (5.46, 6.74)	12,546.9	320.4	1.46 (0.90, 2.02)	12,495.3	297.9	-0.53 (-1.08, 0.03)	1.69 (1.58, 1.80)
75–79	10,464.7	1,194.7	7.49 (6.84, 8.15)	13,861.8	896.0	4.08 (3.53, 4.63)	14,855.2	125.4	-0.49 (-0.99, 0.02)	3.41 (3.30, 3.52)
80–84	9,210.2	1,131.1	8.04 (7.34, 8.74)	13,292.5	1,291.2	6.32 (5.75, 6.89)	15,942.6	549.7	2.01 (1.51, 2.51)	5.40 (5.29, 5.51)

85+	6,485.8	1,109.9	11.41 (10.55, 12.28)	10,048.5	1,184.7	7.64 (6.98, 8.31)	12,439.2	691.7	3.56 (2.98, 4.14)	6.49 (6.36, 6.62)

Abbreviations: APC, annual percent change; CI, confidence interval.

Prevalence expressed per 1 000 000 population.

We estimated the annual average difference with 95% confidence intervals in the prevalence proportions of maintenance dialysis in each age group in 2002–2006, 2007–2011, and 2012–2016 by a generalized linear model with a log-linear link assuming a Poisson distribution. Then, we calculated the estimated annual percent change (APC) as estimated APC=[Exp(estimated annual average difference)-1]×100.

Table S6. The period average duration of maintenance dialysis in the overall and age-specific male groups

	2002–2006			2007–2011			2012–2016		
	P	I	P/I (95% CI)	P	I	P/I (95% CI)	P	I	P/I (95% CI)
Age group, y									
00–04	1.3	1.6	-	0.4	0	-	1.5	1.1	1.4 (0.1–5.8)
05–09	8.8	3.0	2.9 (1.3–5.5)	10.8	3.1	3.5 (1.6–6.1)	10.7	3	3.6 (1.7–6.3)
10–14	26.1	5.9	4.4 (2.9–6.5)	19.7	4.8	4.1 (2.5–6.3)	19.6	4.6	4.3 (2.6–6.6)
15–19	82.8	14.3	5.8 (4.6–7.2)	69.8	13.1	5.3 (4.1–6.7)	60.4	13.9	4.3 (3.3–5.6)
20–24	169.2	27.8	6.1 (5.2–7.1)	175.2	23.2	7.6 (6.5–8.7)	160.7	23.4	6.9 (5.8–8.0)
25–29	355.8	50.4	7.1 (6.3–7.8)	339.5	46.4	7.3 (6.6–8.1)	330.1	46.3	7.1 (6.4–7.9)
30–34	636.6	80.9	7.9 (7.3–8.5)	647.8	84.5	7.7 (7.1–8.3)	622.2	76	8.2 (7.6–8.9)
35–39	1,049.0	135.5	7.7 (7.3–8.2)	1,092.8	138.2	7.9 (7.4–8.4)	1,137.3	144.6	7.9 (7.4–8.3)
40–44	1,492.6	205.1	7.3 (6.9–7.7)	1,707.1	202.7	8.4 (8.0–8.8)	1,874.4	238.6	7.9 (7.5–8.2)
45–49	2,281.0	366.2	6.2 (6.0–6.5)	2,553.6	349.6	7.3 (7.0–7.6)	2,808.1	377.8	7.4 (7.2–7.7)
50–54	3,567.0	625.3	5.7 (5.5–5.9)	3,964.6	581.1	6.8 (6.6–7.0)	4,200.8	569.7	7.4 (7.2–7.6)
55–59	5,293.7	996.7	5.3 (5.2–5.5)	6,065.4	919.5	6.6 (6.4–6.8)	6,212.0	859.6	7.2 (7.0–7.4)
60–64	6,322.0	1,264.9	5.0 (4.9–5.1)	8,472.2	1,371.10	6.2 (6.0–6.3)	8,711.4	1,258.20	6.9 (6.8–7.1)
65–69	7,520.3	1,499.9	5.0 (4.9–5.1)	9,407.8	1,575.40	6.0 (5.9–6.1)	11,625.4	1,741.90	6.7 (6.6–6.8)
70–74	7,344.4	1,637.3	4.5 (4.4–4.6)	10,657.3	2,013.10	5.3 (5.2–5.4)	12,353.6	2,059.90	6.0 (5.9–6.1)
75–79	7,198.8	1,740.7	4.1 (4.0–4.2)	10,378.6	2,192.50	4.7 (4.6–4.8)	13,501.6	2,632.70	5.1 (5.0–5.2)
80–84	7,284.8	1,866.9	3.9 (3.8–4.0)	10,039.2	2,457.10	4.1 (4.0–4.2)	13,138.8	2,873.30	4.6 (4.5–4.7)
85+	5,617.3	1,878.5	3.0 (2.9–3.1)	8,833.8	2,442.00	3.6 (3.5–3.7)	11,459.3	2,902.90	3.9 (3.9–4.0)

Abbreviations: CI, confidence interval; I, incidence; P, prevalence.

The 95% confidence intervals (CIs) on the P/I were estimated from the 95% CI on P assuming a Poisson distribution of prevalence.

The variance of log P, V(log P) = 1/Number of maintenance dialysis cases.

Table S7. The period average duration of maintenance dialysis in the age-specific female groups

	2002–2006			2007–2011			2012–2016		
	P	I	P/I (95% CI)	P	I	P/I (95% CI)	P	I	P/I (95% CI)
Age group, y									
00–04	4.1	1.0	4.1 (1.1–10.2)	5.8	2.1	2.8 (0.9–5.9)	2.5	0.4	6.3 (1.0–20.0)
05–09	5.4	1.6	3.4 (1.0–7.3)	11.0	2	5.5 (2.8–9.9)	5.3	0.8	6.6 (2.0–14.6)
10–14	25.6	5.2	4.9 (3.2–7.2)	15.8	4.9	3.2 (1.8–5.2)	21.9	3.4	6.4 (3.9–9.6)
15–19	58.0	10.2	5.7 (4.3–7.4)	60.6	11.6	5.2 (4.0–6.7)	49.6	10.4	4.8 (3.5–6.3)
20–24	120.6	22.8	5.3 (4.4–6.3)	142.7	25	5.7 (4.8–6.7)	132.3	22	6.0 (5.0–7.1)
25–29	317.1	51.7	6.1 (5.5–6.8)	276.7	42.5	6.5 (5.8–7.3)	292.1	41.2	7.1 (6.3–7.9)
30–34	533.3	65.8	8.1 (7.4–8.8)	573.8	74.2	7.7 (7.1–8.4)	505.2	65.2	7.7 (7.1–8.5)
35–39	869.5	103.4	8.4 (7.9–9.0)	865.2	88.5	9.8 (9.1–10.4)	912.3	96	9.5 (8.9–10.1)
40–44	1,574.7	203.9	7.7 (7.3–8.1)	1,381.8	148.4	9.3 (8.8–9.8)	1,308.0	140.8	9.3 (8.8–9.8)
45–49	2,682.3	348.5	7.7 (7.4–8)	2,420.0	233	10.4 (10.0–10.8)	2,022.9	183.7	11.0 (10.5–11.5)
50–54	4,080.4	553.0	7.4 (7.2–7.6)	3,968.3	396.9	10.0 (9.7–10.3)	3,328.3	305.8	10.9 (10.5–11.3)
55–59	5,917.3	841.5	7.0 (6.9–7.2)	5,810.6	604.4	9.6 (9.4–9.9)	5,227.8	489.7	10.7 (10.4–11)
60–64	7,009.1	1,162.0	6.0 (5.9–6.2)	8,437.2	1,048.40	8 (7.9–8.2)	7,458.3	774.3	9.6 (9.4–9.9)
65–69	8,981.0	1,686.3	5.3 (5.2–5.4)	9,971.0	1,483.90	6.7 (6.6–6.9)	10,786.4	1,346.00	8.0 (7.9–8.2)
70–74	10,567.8	2,117.5	5.0 (4.9–5.1)	12,546.9	2,034.90	6.2 (6.1–6.3)	12,495.3	1,751.90	7.1 (7.0–7.3)
75–79	10,464.7	2,403.7	4.4 (4.3–4.4)	13,861.8	2,634.60	5.3 (5.2–5.3)	14,855.2	2,472.70	6.0 (5.9–6.1)
80–84	9,210.2	2,350.8	3.9 (3.8–4.0)	13,292.5	2,919.90	4.6 (4.5–4.6)	15,942.6	3,125.80	5.1 (5.0–5.2)
85+	6,485.8	1,907.1	3.4 (3.3–3.5)	10,048.5	2,602.90	3.9 (3.8–3.9)	12,439.2	2,868.00	4.3 (4.3–4.4)

Abbreviations: CI, confidence interval; I, incidence; P, prevalence.

The 95% confidence intervals (CIs) on the P/I were estimated from the 95% CI on P assuming a Poisson distribution of prevalence.

The variance of log P, V(log P) = 1/Number of maintenance dialysis cases.

Table S8. The period mortality rates of maintenance dialysis and the average annual percentage change in the age-specific male population

	2002–2006			2007–2011			2012–2016			All observed years
	Number of cases	Patient-year of observation	Rate	Number of cases	Patient-year of observation	Rate	Number of cases	Patient-year of observation	Rate	Estimated APC (95% CI)
Age group, y										
00–04	2	3.5	57.1	0	1.5	0.0	0	3.5	0.0	-11.34 (-14.49, -8.06)
05–09	6	36.0	16.7	2	36.0	5.6	0	26.5	0.0	-24.5 (-28.93, -19.8)
10–14	4	110.0	3.6	7	79.0	8.9	4	64.5	6.2	-0.99 (-5.59, 3.84)
15–19	8	357.5	2.2	6	296.5	2.0	5	244.0	2.0	-2.60 (-10.18, 5.62)
20–24	10	821.5	1.2	11	749.0	1.5	7	675.0	1.0	-1.43 (-11.28, 9.52)
25–29	34	1,740.5	2.0	18	1,677.5	1.1	17	1,390.0	1.2	-5.42 (-14.46, 4.58)
30–34	61	2,896.5	2.1	58	3,149.5	1.8	66	3,075.5	2.1	1.72 (-6.31, 10.44)
35–39	119	4,933.5	2.4	135	4,945.0	2.7	149	5,501.5	2.7	1.18 (-5.89, 8.79)
40–44	249	6,523.5	3.8	237	7,980.5	3.0	261	8,284.0	3.2	-2.8 (-8.81, 3.61)
45–49	495	9,168.5	5.4	472	11,965.0	3.9	505	12,853.5	3.9	-4.39 (-9.54, 1.06)
50–54	871	12,170.5	7.2	904	16,878.5	5.4	1,022	19,256.5	5.3	-4.55 (-8.99, 0.11)
55–59	1,116	11,381.0	9.8	1,574	21,552.0	7.3	1,865	25,662.0	7.3	-4.55 (-8.37, -0.58)
60–64	1,354	10,906.0	12.4	1,936	18,551.0	10.4	2,804	29,603.5	9.5	-3.96 (-7.31, -0.50)
65–69	1,736	11,195.5	15.5	2,000	16,374.5	12.2	2,841	23,764.0	12.0	-3.86 (-6.89, -0.73)
70–74	2,008	9,789.5	20.5	2,447	15,044.0	16.3	2,983	19,322.5	15.4	-4.13 (-6.77, -1.42)
75–79	2,001	8,054.5	24.8	2,483	12,221.0	20.3	3,207	15,829.5	20.3	-3.22 (-5.60, -0.79)
80–84	1,437	4,557.0	31.5	2,349	8,636.0	27.2	3,040	11,427.5	26.6	-1.88 (-4.01, 0.30)

85+	867	1,818.5	47.7	1,767	4,383.5	40.3	3,344	8,389.0	39.9	-1.61 (-3.36, 0.17)

Abbreviations: APC, annual percent change; CI, confidence interval.

Mortality rate expressed per 100 person-years.

We estimated the annual average difference with 95% confidence intervals in the mortality rate of maintenance dialysis in each age group in 2002–2016 by a generalized linear model with a log-linear link assuming a Poisson distribution. Then, we calculated the estimated annual percent change as estimated APC=[Exp(estimated annual average difference)-1]×100.

Table S9. The period mortality rates of maintenance dialysis and the average annual percentage change in the age-specific female population

	2002–2006			2007–2011			2012–2016			All observed years
	Number of cases	Patient-year of observation	Rate	Number of cases	Patient-year of observation	Rate	Number of cases	Patient-year of observation	Rate	Estimated APC (95% CI)
Age group, y										
00–04	1	11.5	8.7	1	14.0	7.1	1	6.5	15.4	0.81 (-2.78, 4.53)
05–09	0	18.0	0.0	3	30.5	9.8	1	15.5	6.5	-2.43 (-7.27, 2.67)
10–14	2	89.5	2.2	4	61.0	6.6	1	66.0	1.5	-3.25 (-9.51, 3.44)
15–19	6	207.5	2.9	8	236.5	3.4	2	181.0	1.1	-11.49 (-18.18, -4.27)
20–24	11	511.5	2.2	6	553.5	1.1	7	521.5	1.3	-8.59 (-16.95, 0.60)
25–29	28	1,359.5	2.1	32	1,360.0	2.4	18	1,152.0	1.6	-4.83 (-12.37, 3.35)
30–34	44	2,148.0	2.0	70	2,788.5	2.5	55	2,520.5	2.2	0.82 (-6.65, 8.88)
35–39	75	3,655.0	2.1	75	3,919.5	1.9	106	4,494.5	2.4	-0.38 (-8.00, 7.87)
40–44	160	6,759.0	2.4	145	6,560.0	2.2	164	5,970.0	2.7	0.57 (-6.60, 8.30)
45–49	320	10,645.5	3.0	306	11,594.0	2.6	286	9,596.0	3.0	-2.42 (-8.82, 4.42)
50–54	613	13,885.0	4.4	604	17,402.5	3.5	550	15,939.0	3.5	-3.38 (-9.01, 2.59)
55–59	902	12,952.5	7.0	1,100	21,433.0	5.1	1,090	22,741.5	4.8	-5.14 (-9.68, -0.36)
60–64	1,207	12,847.0	9.4	1,481	19,564.0	7.6	1,850	27,133.5	6.8	-4.67 (-8.54, -0.65)
65–69	1,833	14,737.5	12.4	1,901	19,345.0	9.8	2,275	24,291.5	9.4	-4.01 (-7.39, -0.50)
70–74	2,335	13,715.0	17.0	2,694	20,731.5	13.0	2,871	22,982.0	12.5	-4.51 (-7.42, -1.50)
75–79	2,178	9,792.5	22.2	3,082	17,018.0	18.1	3,806	22,019.0	17.3	-3.68 (-6.20, -1.10)
80–84	1,555	5,176.0	30.0	2,592	10,495.0	24.7	3,782	15,978.5	23.7	-3.38 (-5.55, -1.15)

85+	1,027	2,336.5	44.0	2,175	5,580.0	39.0	3,865	9,992.0	38.7	-2.15 (-3.92, -0.35)

Abbreviations: APC, annual percent change; CI, confidence interval.

Mortality rate expressed per 100 person-years.

We estimated the annual average difference with 95% confidence intervals in the mortality rate of maintenance dialysis in each age group in 2002–2016 by a generalized linear model with a log-linear link assuming a Poisson distribution. Then, we calculated the estimated annual percent change as estimated APC=[Exp(estimated annual average difference)-1]×100.

Table S10. Use of relevant services in patients with kidney failure in 2002–2016

	Calendar year														
	2002	2003	2004	2005	2006	2007	2008	2009	2010	2011	2012	2013	2014	2015	2016
No. using a hospice advisory service before death$	-	-	-	-	-	-	-	0	0	0	0	157	231	423	635
No. receiving kidney transplantation before dialysis	20	13	18	19	20	24	15	33	30	23	25	35	33	18	30

Abbreviation: No., number.

Data from the Annual Report of Kidney Disease in Taiwan.

Hospice advisory services for patients with chronic kidney disease started reimbursed by Taiwan National Health Insurance in 2009.
